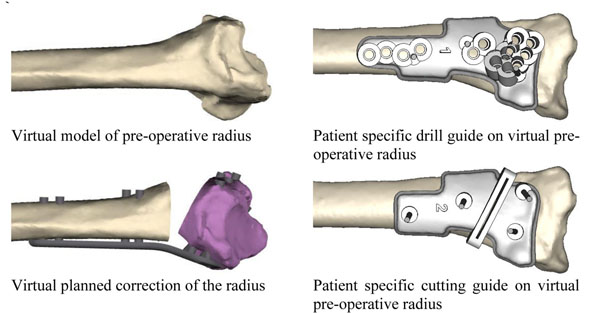# Osteotomy and grafting of the distal radius using virtual planning and patient specific guides

**DOI:** 10.1186/1753-6561-9-S3-A96

**Published:** 2015-05-19

**Authors:** Stephen Kemp

**Affiliations:** 1 Hunter Hand and Upper Limb Surgery, New South Wales, Australia

## 

Corrective osteotomy of the distal radius for malunion has always been a challenging procedure. Deformity involves length, angulation and rotation. Planning an osteotomy on plain x-rays is difficult and in-exact. Reproducing that planned osteotomy in the operating theatre is even more difficult.

Attempts to improve accuracy with the use of CT scanning and even the production of models have met with varying success. More recently however, software (Materialise N.V, Belgium) has been developed which allows the generation of virtual 3-D models of the bones of both forearms from a CT scan following a specific protocol. These models can be manipulated in a virtual environment. The contralateral model can be mirrored and superimposed on the malunion. Following a virtual osteotomy the fragment of the malunited radius can be manoeuvred into a desired post-operative position using the normal radius as a template for correction. Virtual models of a specific plate and corresponding screws can be fitted into the best position.

Reverse engineering this virtual plan allows a series of patient specific guides to be designed and produced using additive manufacturing technology. All the screw holes can be drilled first followed by the osteotomy. The soft tissues are released as necessary and the plate applied.

In comparison to experiences with similar cases done conventionally, the observed accuracy of the surgery is quite astounding. The correction is achieved to within millimetre tolerance. Operative time is reduced. The real strength of this approach is its versatility. The surgeon can sit down with an engineer over the internet and actively participate in the planning of each surgery. In simple corrections this can be straightforward, however more significant corrections require careful assessment and a flexible approach.

The technology will be presented from a surgeon's perspective and three cases used to highlight the versatility of the approach.

**Figure 1:**